# School Nurses’ Perceptions, Learning Needs and Developmental Suggestions for Mental Health Promotion: Focus Group Interviews

**DOI:** 10.3390/ijerph17249503

**Published:** 2020-12-18

**Authors:** Minna Anttila, Milla Ylitalo, Marjo H. Kurki, Kirsi Hipp, Maritta Välimäki

**Affiliations:** 1 Department of Nursing Science, University of Turku, 20014 Turku, Finland; minna.anttila@utu.fi (M.A.); milla.ylitalo@laurea.fi (M.Y.); mhkurk@utu.fi (M.H.K.); kirsi.hipp@utu.fi (K.H.); 2 Laurea University of Applied Sciences, 01300 Vantaa, Finland; 3 Itla Children’s Foundation, 00180 Helsinki, Finland; 4 Xiangya School of Nursing, Central South University, Changsha 410013, China

**Keywords:** adolescent, focus group, mental health, health promotion, school nurse

## Abstract

School nurses have a key role in promoting the mental health of adolescents at school. However, there is still a lack of comprehensive understanding of the role and experiences of school nurses as they promote mental health at schools. A qualitative research design employing focus group interviews was used. School nurses (*n* = 21) were purposively sampled from one city in Southern Finland. The data were analyzed using inductive content analysis, resulting in seven categories describing school nurses’ perceptions, needs and suggestions for development of mental health promotion in school health care. School nurses perceived health care at school as a low-threshold service. Mental health problems are often first identified by a school nurse. However, school nurses felt that extra effort is needed to recognise mental health problems, build trusting relationships, and motivate adolescents to attend regular health check-ups. Specific core learning competencies such as communication skills, being present, keeping confidentiality, and the ability to motivate adolescents to regularly visit the school health clinic are needed. However, school nurses often lack basic resources for mental health promotion. The areas of mental health development included cooperation with stakeholders and parents, and development of anonymous, easy-access services. It is important that school nurses have the skills needed and enough resources to fulfil their demanding tasks in school health care services.

## 1. Introduction

Mental health problems account for 16% of the global burden of disease and injury among 10–19-year-olds [1]. The number of psychiatric and neurodevelopmental diagnoses among minors has increased internationally [2,3]. Over the past ten years in Finland, these diagnoses in specialized mental health services have increased by 50% among 12–17-year-olds [4]. At the same time, mental health problems are still underdiagnosed and undertreated among students [5]. It has been estimated that only 10–15% of adolescents receive adequate treatment from health care services [6]. As adolescence is a critical developmental period when risk for mental disorder increases [7], early identification of symptoms of depression and anxiety is important [8]. If untreated, these mental health problems may extend to adulthood and impair physical health, work performance, and quality of life [1].

Schools are good settings for facilitating early recognition and signposting for treatment of mental disorders and promoting evidence-based mental health interventions [9]. It has previously been proposed that mental health services should be provided in community settings and especially in schools, where adolescents spend time [5,10,11,12]. Mental health promotion in schools is cost-effective [13,14], and most schools are staffed with multidisciplinary personnel who have the potential to identify problems and offer psychosocial support [15]. For example, in Finland, adolescents’ annual health screening and counselling at school are regulated by law [16] and national guidelines [17,18]. Eighth graders (on average aged 14 years) are especially screened for depression. Adolescents and their guardians are also invited to join a meeting organized by school nurses to map any health issue related to adolescent wellbeing [19]. School nurses in Finland are part of a multidisciplinary welfare team at school [20,21]. If any concerns related to adolescents’ mental health arise, the school nurse can discuss them with the adolescent, family, teachers, psychologists, and a doctor at the school, and a referral can be made to psychiatric services [16]. Still, adolescents are not so active in seeking help from professionals [22]. They lack knowledge about mental disorders, and often do not believe in their own capabilities [23,24]. They also lack the confidentiality to interact with professionals [25] and access care [26]. They often experience stigma and shame tied to mental health problems [27]. However, based on the Finnish register study [28], a larger number of adolescents in Finland are diagnosed with depression in specialized services than before. This indicates an increase in identification in primary health care, such as school health care, and more positive attitudes towards mental health problems and treatment. There is still a great need in primary health care for improvement in access to mental health care, as seen over the last ten years. Especially adolescents’ preventive mental health care and evidence-based interventions for depression and anxiety in school environments are highlighted in a new national Future Health and Social Services Centres program [29]. Further, the School Welfare Act (1287/2013) specifies that school nurses, counselors and psychologists must provide students with immediate and low-threshold access to their services [20].

School nurses consider mental health to be an important part of their work and that their role should be to focus on health promotion, assessment and early intervention activities [30]. Key components of school nurses’ workload come from mental health promotion [30,31,32] and preventive actions regarding anxiety [5], depressive symptoms [33] and self-destruction [34]. Even though school nurses have professional competence in mental health [35,36], studies have identified a lack of knowledge among school nurses regarding mental health promotion as well as a need for further training [5,10,32,37]. A global overview of school health services from 102 countries showed that mental health may not be given sufficient consideration in routine service provisions [38]. Therefore, it is important that school nurses have support and consultation from local mental health specialists [36]. In addition, literature highlights the importance of their collaboration with families and other school personnel [10,39,40].

Despite promotive mental health interventions having been developed for schools [39,40,41,42], there is still a lack of experience among school nurses in how to realize mental health care in school setting [32] and be part of collaborative mental health care [10]. Exploring the views of school nurses is essential for identifying purposeful practices and development areas for mental health promotion in school settings [15] when they face, for example, an increasing number of adolescents suffering from neurodevelopmental diagnoses [2,3] or culturally diverse adolescents with mental health problems [43]. In this study, we aimed to describe school nurses’ perceptions, individual learning needs and developmental suggestions for mental health promotion among adolescents at school. Therefore, the following research questions were asked: (1) What are school nurses’ perceptions of mental health promotion in health care at school? (2) What are the needs of school nurses regarding knowledge, skills and support in promoting adolescents’ mental health at school?; and (3) How do school nurses suggest that the promoting of adolescents’ mental health and well-being at school could be developed?

## 2. Materials and Methods

### 2.1. Design

A qualitative study design with focus group interviews and content analysis [44,45] was used as a suitable method to describe individual perceptions, needs and developmental suggestions [46] of school nurses working in a school setting. They form a homogenous group [47] in Finnish health care services. Consolidated criteria for reporting qualitative studies (COREQ) [48] were followed.

### 2.2. Study Setting and Participants

The Finnish education system [49] has two compulsory levels: one-year of pre-primary education for 6-year-olds, and basic education (grades 1–9, aged 7–16). Basic education includes elementary school (grades 1–6) and lower secondary schools (grades 7–9). These compulsory levels are government-funded and therefore free of charge. Also, pupils with special needs mostly attend basic education with the support of auxiliary services. After basic education, pupils can choose post-compulsory upper secondary school, which constitutes either general or vocational education for students 16–19 years old. In this paper, we describe the health care for adolescents (aged 12–19) in lower secondary schools (aged 12–15, grades 7–9) and post-compulsory upper secondary schools (aged 16–19). Health care at school is part of the primary health care support adolescents receive to support learning and physical, psychological and social welfare.

Participants were school nurses working in lower secondary schools and post-compulsory upper secondary schools. School nurses in Finland are employed by the local community and their services are free of charge to the adolescents. School nurses are trained as public health nurses, an education program that generally takes four years at a university of applied sciences [49]. In Finland, school nurses are specialized in health promotion including mental health and preventive work, but they also offer treatment. Public health nurses are specialists in both in nursing and public health. As public health nurses, they support and guide individuals, families and communities in issues related to health and life management at different life stages.

### 2.3. Recruitment

School nurses were recruited using purposive sampling, which allowed for finding common features of the study [50]. The head school nurse in one city (population about 200,000) was approached first, and she was introduced the study. The selected city is one of the largest in Finland and was selected as it represented a typical city in Southern Finland.

The head school nurse informed all the school nurses working at the lower secondary schools (*n* = 37) and post-compulsory upper secondary schools (*n* = 16) about the study, and a face-to-face meeting was proposed. She also encouraged the school nurses to join the study. If a school nurses agreed to participate, their contact information was collected, and a researcher approached them by mail.

The school nurses were eligible to participate if they worked in health care services at a school with adolescents (aged 12–19 years), had the opportunity to participate in the research during working hours, were able to read, write, and speak Finnish, and signed a voluntary informed consent form [51]. A total of 21 school nurses out of 53 were enrolled regardless of their work experience or qualification certificate. All of them met the inclusion criteria. No relationship was established with them prior to study commencement.

### 2.4. Ethical Considerations

The study is approved by the Health and Social Welfare Department of the city (Dno SOSTER 795/2013/092) in which the schools were located. Approval from ethics committee was not obtained because the study did not involve patients, and the participation of school nurses was voluntary [52,53]. No incentives were given to the participants, and the data was handled in confidence.

### 2.5. Focus Group Interviews

The data were collected using focus group interviews [54]. This method was selected as it is suitable for providing insights into participants’ perceptions of complex issues through an active interaction [46]. A semi-structured interview guide was developed for the study, based on existing literature [30,33] and the Theory of Planned Behavior [55]. This theory explains how peoples’ behavioral intentions are influenced by their attitudes and beliefs, and how their behavior is based on expected outcomes, subjective norms, perceived control and social pressures. In this study we describe school nurses’ perceptions of mental health promotion at school, their individual learning needs to promote adolescent mental health at school, and their developmental suggestions for mental health promotion at school. The interview guide was tested with two registered school nurses and revised according to their responses and comments [56].

The questions were open-ended, allowing the school nurses to describe their perceptions, needs and developmental suggestions in addressing mental health care in school [57]. An open-ended approach allowed the interviewer to pose more detailed questions based on school nurses’ responses [58]. The interview questions were formulated with the aim of producing answers to the research questions, mentioned above.

The background information of the participants (age, gender, working experience, workstation and mental health training) was collected with a paper questionnaire.

### 2.6. Procedure

The interviews at two schools were conducted between May and September 2013. At the beginning of the interviews, the researcher introduced herself and explained how she was doing the research as part of her MNSc studies and based on her working experience in the field of mental health care. Oral and written information about the study was provided, and written informed consent was obtained from the participants [59].

The interviews consisted of three focus groups. The timing of the focus group interviews was decided based on participants’ schedules, and the interviews were organized to coincide with the school nurses’ usual team meetings to avoid adding to their inconvenience. There were five to eight participants in each focus group. According to Stewart, Shamdasani, and Rook [60], a group size of six to ten participants, with three to five groups, ensures data saturation.

The interviews were audio-recorded with the participants’ permission. In addition, the researcher wrote descriptive field notes of her observations during the interviews [58], and no interviews were repeated. The length of the interviews ranged 60–68 min. One of the authors, a MNSc student (M.Y.), conducted all of the interviews, and there was one additional facilitator present (a registered nurse), who made notes and asked further questions when there was a need to clarify unclear statements and confirm the accuracy of the interviewers’ interpretations [60].

### 2.7. Data Analysis

Data was analyzed using inductive content analysis [45,61] to answer the three main research questions. First, the audio-recorded interviews were transcribed verbatim [62] in a Word document. Second, the audio-recordings were listened to, and the text was read several times and reviewed by the researchers. Third, the researchers chose statements to be meaning units, and these were identified and summarized into codes. Codes were further combined based on their similarity into sub-categories, which reflected and were named by their content. Finally, sub-categories were grouped to develop the main categories [45,61]. Categories comprised themes derived from the data and responded to the specific research questions [61]. The analysis was conducted by following researchers: MNSc student M.Y., Ph.D. student M.K., and Ph.D. M.A.

## 3. Results

### 3.1. Description of the Participants

In total, 21 school nurses participated in three focus group interviews at school. All participants were female, aged between 25 and 63 years. The length of their working experience as a public health nurse varied from six months to 35 years, and they had worked in school nursing from six months to 28 years. Out of the school nurses, 15 currently worked in school health care in lower secondary schools and six in student health care in post-compulsory upper secondary schools. Fifteen out of 21 school nurses had had the possibility to participate in continuing education.

### 3.2. School Nurses’ Perceptions of Adolescents’ Mental Health Promotion at School

School nurses’ perceptions of adolescents’ mental health promotion included three categories (Table 1). First, school nurses discussed their role as a mental health promoter at school. The school nurses considered health care at school as a low-threshold environment, where all adolescents are met and where it is easy for adolescents to seek help. It may often be the place of contact in which adolescents first talk about their problems or where their problems are first noticed. The work of school nurses is increasingly turning to promoting and treating mental health problems at school; their task is to try to identify and screen mental health problems and support adolescents’ mental well-being. Discussions with adolescents about everyday events was emphasized in their job description. Although the school nurses considered the school health care setting as a place for promoting adolescents’ mental health, and stated they would like to do so more, it was not always possible in clinical practice because of a lack of time and recourses.

Second, the school nurses discussed the mental health problems they had observed among adolescents at school. The school nurses thought that the occurrence and severity of mental health problems in this age group is increasing. These problems also pose a risk to other adolescents and can have seasonal variation. School nurses identified gender-related differences on how mental health problems appear; boys’ problems are more difficult to identify and treat from a nurses’ point of view.

Third, the school nurses discussed their attitudes as well as the attitudes of adolescents and their families regarding mental health. The school nurses found it important to take care of mental well-being and believed that treatment for mental health problems is worthwhile. They also trusted in their own ability to help adolescents with mental health problems but highlighted that they are not psychotherapists. They found adolescents’ attitudes positive, but their commitment to treating problems ambivalent and challenging. Moreover, parents may have more negative attitudes than adolescents towards mental health problems and treatment, which also negatively affects the adolescents’ commitment to treatment.

### 3.3. School Nurses’ Need for Knowledge, Skills and Support at School

School nurses’ need for knowledge, skills and support constituted two categories (Table 2). First, the school nurses expressed needing more training in adolescents’ mental development and mental health problems in general, even though they were quite satisfied with the education they had. In addition, the school nurses stated that they needed individual and accurate information about the mental status of the adolescents, as well information about how they were managing in the daily school environment. The school nurses also wished for more information about the adolescents’ background and family to better understand the situations. Some knowledge about the care system was also needed. The school nurses emphasized the importance of professional communication, by which they meant the ability to ask about things and bring up issues in discussions, a genuine ability to be present, the ability to deal with each adolescent in a fair way and accept the adolescent for who they are. Particularly important to the school nurses was the ability to gain the trust of the adolescents at school and to motivate the adolescents to regularly visit the school nurse if they had established mental health needs. In addition, they highlighted the importance of practical skills, such as how to encounter mental health problems in practice and instructions for when to be particularly concerned about symptoms.

Second, the school nurses needed support at school. Clinical supervision was mentioned in the interviews. However, school nurses valued peer support more, as well as the possibility to share difficult cases as soon as possible. The school nurses believed their most important working partners were school psychologists, welfare officers, teachers, doctors, and the welfare team. School nurses wished for more open collaboration, better information flow, sharing of responsibilities, confidence as professionals, and feedback from others, such as specialized care and youth specialist psychiatric care. The school nurses thought their manager’s support was important at school, but they thought that, in practice, managers were too distant from basic, everyday work. From a manager, they expected a good understanding of how their working hours are spent. This meant that they felt much of their time-consuming work remained invisible and was not taken into account when allocating resources. The school nurses considered it essential that adolescents’ families support the treatment. Cooperation with families was thus seen as very important, but sometimes also challenging. The school nurses identified a need for parental involvement in their child’s treatment, not least because parents have a significant role in how an adolescent’s care is organized. Indeed, families can be supportive of the treatment, or deny care.

### 3.4. School Nurses’ Developmental Suggestions for the Future

This theme formed two categories (Table 3). First, the school nurses agreed that more variation in services should be made available for adolescents. The school nurses expressed the need for services where an adolescent can have a conventional conversation or anonymous discussions. By ‘easy access’ they meant that services should be readily available and possible to obtain from one location, without being sent from one place to another. The school nurses also felt that services should include online help. The possibility to take part in group activities was also mentioned.

Second, the school nurses stated that they need more resources, such as time, to promote adolescents’ mental well-being at school. They considered promoting mental health to be a very important part of their work, but one which is not always visible, and therefore, there is not enough time available for it. They also expressed needing more resources for developing cooperation with other professionals and engaging parents in adolescent care.

## 4. Discussion

In this paper, we describe school nurses’ perceptions, needs and developmental suggestions to promote adolescents’ mental health and well-being in school environments. Further, we discuss the role of school nurses, and their possibilities and competencies to respond to the recent mental health strategies at school. Our findings support previous studies that show that school nurses have a key role in promoting adolescents’ mental health in a school setting [31,36]. School nurses found health care at school to be a place to promote adolescents’ mental health, which is in line with the international and national mental health strategy that highlights early-intervention approaches within primary health care services [9,12]. Health care at school may also be less stigmatizing than mental health clinics [10]. According to Haddad and Tylee [63], about half of adolescents experience stigmatization regarding mental health problems and consider treatment to have negative consequences, such as gossips, avoidance and under-estimation in their daily life. Also, school nurses described their uncertainty in documenting mental health issues out of concern that it may stigmatize students [64].

The school nurses felt that promoting adolescents’ mental health is an important part of their work. This finding is supported by Pryjmachuk et al. [30], who found that school nurses highly value the mental health side of their work and spend a notable amount of time supporting adolescents with mental health problems. However, even though the school nurses stated that their basic work covers all kind of health promotion, they thought that they had to take care the mental health of adolescents more often than any other kind [30]. It has been reported that only 10–15% of adolescents with mental health problems receive the required treatment from health care services [22]. Difficulties in interaction with professionals have been described to be barrier to help seeking, especially at school [25]. On the other hand, Kivimäki et al. [65] found that Finnish adolescents reported that accessing a school nurse is easy, even though this varied between schools. The open-door policy of school nurses is important for adolescents as they are often the only health professionals at school that adolescents can go to without a scheduled appointment [65]. Easy access mental health services have also been recommended in national [12] and international [9] mental health strategies.

On an individual level, our study showed that school nurses were engaged in mental health promotion at school and that they trusted their own ability to help adolescents. This may be due to the fact that out of 21 school nurses, 15 (71%) had some continuing education concerning mental health. Haddad et al. [66] also found in their intervention study that school nurses benefit from short courses on depression. Their knowledge on depression has been shown to improve and their confidence shown to increase [66]. Our results strengthen the evidence [67] that school nurses struggle to recognize and treat mental health problems among boys. Noteworthily, all the participants were female, reflecting the female-dominated occupation of school nurses. Male school nurses could have different perceptions and preferences regarding boys’ mental health. In addition to gender, age and work experience may influence the ability of school nurses to promote mental health. Future studies should focus on how school nurses could be adequately supported throughout their careers.

Contrary to previous studies [5,10,31,32,36,37], the school nurses in our study thought that a lack of resources, rather than a lack of training, is the barrier for promoting and treating mental health problems on an institutional school health care level. This finding is promising, as it suggests that adolescents’ mental health promotion can be done in the health care setting at school with relatively little additional training and with the support of special care. To implement the mental health promotion within primary health care, it is urgent to ensure adequate resources for this work [9,12]. If depressive disorders, for example, are systematically screened at schools, there needs to be easily accessible treatment for adolescents with mild or moderate depression. The school health care setting is not the place in which all mental health work can or should be done [30].

The school nurses emphasized the importance of multiprofessional cooperation as well as collaboration with parents to support their work at school. Earlier studies have also found that a lack of confidence needs to be managed, and it is important that school nurses are supported by local specialist mental health teams [10,30,36]. Our study did not reveal any lack of confidence, but the school nurses hoped for more open collaboration and better information flow from specialized care. Previously, interprofessional tensions and power dynamics have been noticed, as in a study by Reinke et al. [68], where teachers viewed school psychologists to have a primary role in most aspects of mental health service delivery, such as conducting screening and behavioral assessments, monitoring adolescent progress, and referring them to school-based or community services. Coordinated collaboration and use of systematic indicators have been stated to be critical in carrying out mental support for adolescents [9,12], and there is a need for further investigation into school nurses’ networks, too. Especially in modern-day Europe, school nurses need adequate training for promoting mental health among culturally diverse adolescents, a group that has increased in number and that has a high prevalence of mental health problems [43].

Fostering of adolescents’ mental health and well-being is an investment in the future. Good mental health enables social relations, effective learning, the ability to care for oneself, good physical health, and participation in society [11]. In contrast, problems with mental health are a major cause of disability and may cause enormous suffering for individuals, families and communities [69].

Developmental suggestions that the school nurses expressed for their work at school included, first of all, a need for more easily accessible services for adolescents in the school environment, such as conversations, group activities or online help. The importance of this suggestion has been highlighted in recent years as international and national recommendations support the idea that adolescents’ mental health services should be provided in daily environments [12,70,71]. Inexpensive and minimally time-consuming assessments and interventions are in demand for schools, as it is particularly important to gain the trust of the adolescents and to motivate them to participate in regular visits if they have established mental health needs. School nurses need, for example, skills to do motivational interviews that support adolescents’ self-esteem and enable adolescents to better face their challenges [36]. School nurses also have first-hand knowledge of the adolescents in their school; for example, our study found that mental health problems seem to have seasonal variation and differ between genders. However, even though school nurses would like to promote more adolescents with mental health problems, this is not always possible due to a lack of time and resources. A recent Finnish study by Gyllenberg [72] points out that there are very few mental health services in primary health care—especially evidence-based interventions are lacking in many municipalities. Another challenge is mainstreaming e-mental health interventions, in particular, into health care [9].

### Limitations and Strengths

There are a few limitations as well as strengths concerning the study. First, the sample size of the participating school nurses was relatively small (*n* = 21); nevertheless, it was in line with the guidance of having three to four focus groups [73] each consisting of four to ten participants [56] to obtain enough data. Saturation was achieved as there was repetition in categories among the focus group interviews [44]. On the other hand, the trustworthiness of the findings would have been strengthened if we had returned the transcripts or the key categories to the participants.

Second, the school nurses were all female and currently working in the same city, which complicates generalizing the findings outside of the local context. Only those with an interest in mental health care may have attended the focus groups, which, while assuring that the school nurses participated in the interviews voluntarily, may have produced ungeneralizable results. Moreover, the interviews were conducted in Finnish, necessitating translations of quotations into English, which may have affected the understandability of the quotations and limited their comprehensibility in other countries.

Third, the data collection was completed in 2013; thus, the findings might seem outdated. However, the present study describes the situation at the beginning of 2010s, the decade in which stressful events such as natural disasters, terrorist attacks, and mass shootings occurred. The decade also included advances in individual civil rights, e.g., Black Lives Matter, advances in lesbian, gay, bisexual, transgender and queer or questioning (LGBTQ) rights, and the #MeToo Movement [74]. Therefore, year after year, school nurses need to be competent in their communication skills. They need to ask about things, bring issues into the discussion, be present, handle the adolescent in a fair way, and accept the adolescent for who they are. They need to gain the trust of the adolescent and motivate them to attend regular visits if they have an established mental health need. This can be realized in health care settings at school if it is easy for adolescents to come and seek help, talk about their problems and discuss everyday events.

To conclude these limitations, it can still be said that our research produces strengths and valid information regarding the role of school nurses and possibilities to promote adolescents’ mental health at school, even during changes. Moreover, as there is global variance in school-based health services, especially between low- and high-income countries [75], disseminating knowledge of mental health promotion can support planning and implementing services in schools worldwide.

## 5. Conclusions

School nurses are motivated to support mental health at school as a part of their work in a primary health care setting. From their viewpoint, it is not a lack of training that can hinder promotion and treating adolescents’ mental health problems, but rather a lack of resources. It is therefore important to gain more knowledge on resources schools currently have and what kind of resources are needed. School nurses’ views should also be considered, to identify and implement the most helpful mental health interventions at school and to critically evaluate school-based promotion programs. Research and exchange of experiences are, therefore, needed to further investigate the roles and responsibilities of school nurses, teachers and mental health professionals in mental health promotion prior to the onset of a mental disorder. Good practice models and evidence-based, inexpensive, and minimally time-consuming interventions are of importance to run mental health care in schools.

The school nurses in our study in 2013 were able to evaluate upcoming requirements of their work when we asked their suggestions for developing the promotion of adolescents’ mental health at school in the future. They called for more individual variation in services, such as anonymous services, services that are possible to obtain from one location, online help, and group activities. They also expressed the need for resources such as more time for mental health promotion, cooperation with other professionals and parent engagement in care. However, there were many descriptions in which school nurses were concerned about identifying and providing care for mental health problems at school, even if mental health promotion was the focus of the study. This indicates that the number of mental health problems in health care has increased and is also seen in school nurses’ everyday work life.

### Relevance for Clinical Practice

Since health care at school is uniquely positioned to reach all adolescents, health care should take major responsibility for promoting adolescents’ mental health at school. Therefore, it is important to develop mental health care in the school environment [70,71]. There has been a call for more accessible and less stigmatizing services, compared to those that have already been in use in mental health specialty care [10,27]. Moreover, based on the national recommendation of the Ministry of Social Affairs and Health [29] to implement mental health services into school health care, more research is needed to find out what the effects of this reform are on school nurses’ mental health competence. It is also essential that adolescents’ families support the work and are willing to cooperate. Evidence-based and less time-consuming interventions in schools and easily accessible services are needed in promoting adolescents’ mental health and well-being. Positive aspects of the school nurses responding to modern era mental health needs at school are that they seem to be satisfied with the education they have, and they trust in their own ability to help adolescents with mental health needs.

## Figures and Tables

**Table 1:** Adolescents’ mental health promotion in health care at school.

Categories	Sub-Categories	Excerpts from the Interviews (I 1–3)
School nurse as a mental health promoter at school	Low-threshold care in the everyday environment	*(…) as the treatment should be in the everyday life of the adolescent, close to the everyday life, that it would not always be differentiated to be part of something else, away from adolescents…* (I 2)
	Place for first contact	*(…) we have announced that if there is a problem with coping, then you can come to the school nurse and talk about it…* (I 1)
	Main task of health care at school	*(…) sometimes it feels that they benefit from it, that they come to chat with someone they know, with a school nurse, even if we are not any specialists…* (I 3)
	Lack of resources	*(…) if one had time, one would still emphasize with things a bit differently…* (I 2)
Mental health of adolescents	Increased mental health problems of adolescents	*(…) all in all, this is an area that has continued to grow. If I think about my own long career, it seems that my task becomes more and more to support mental health…* (I 1)
	Differences between genders	*(…) and what I worry about indeed are boys. Girls somehow come more easily and tell, but boys stay, they stay in a position of a step-child in a way, that they do not. Then they show otherwise, they rage, brawl, are absent and everything else. But seldom do they come and are sort of verbally able to tell about these things…* (I 2)
Attitudes toward mental health	Positive attitudes of school nurses	*(…) I would see that we have managed to solve many things there, even through school discussion…* (I 1)
	Positive attitudes of adolescents	*(…) adolescents are beginning to have such attitude that you can talk about how it feels, and about depression and so on…* (I 1)
	Attitudes of families	*(…) I think that older generations, one did not talk about them or they were kept inside a family or were being treated on a certain, small scale…* (I 1)

**Table 2:** School nurses’ need for training and support at school.

Categories	Sub-Categories	Excerpts from the Interviews (I 1–3)
Training needs	General knowledge about mental development	*(…) when we go to the side of the symptoms, and what is part of normal puberty…* (I 2)
	Individual knowledge about mental health problems	*(…) the background, that what happens there in a family, it is also a very essential thing, that one understands the adolescent and his/her behavior…* (I 1)
	Knowledge about care system	*(…) where you are referring to and sort of new views about these systems…* (I 1)
	Communication skills	*(…) you do not have sort of prejudices, pre-ideas related to anything, but it is always individual, so it is such a skill what is needed in recognising and of course, more over in meetings…* (I 2)
	Practical skills	*(…) aid to the screening, sort of what is worrying, what is somehow seen as normal…* (I 2)
Need for support	Clinical supervision	*(…) these always change these (prescription) instructions…* (I 3)
	Peer support	*(…) of course from the school community, psychologist, welfare officer, school doctor…* (I 1)
	Professional collaboration	*(…) and one should also somehow cooperate with the persons in charge of the treatment. And somehow with the school, too, that they could consult, at least something. Openness in it. If they go there and are here, sure some information should be delivered from it…* (I 1)
	Managerial support	*(…) at the moment I would mostly need support from the management, that they, so that they sort of understanding that these things, these are what we talk and shout about. That I even, that the one who does not do this work does not completely understand that it may take an enormous amount of time…* (I 2)
	Family support	*(…) parents do have quite a big role with that child, that do they want the treatment or not…* (I 3)

**Table 3:** School nurses’ developmental suggestions for future promotion of adolescents’ mental health at school.

Categories	Sub-Categories	Excerpts from the Interviews (I 1–3)
Variation in mental health services	Face-to-face conversation	*(…) when s/he settles down, I guess the adolescent feels that there is an opportunity to talk to them…and it is of extremely important place, when they open their mouth…* (I 1)
	Easily accessible interventions	*(…) that one does not need to refer an adolescent all around, from one hatch…* (I 3)
	Online help	*(…) this online service…* (I 1)
	Group activities	*(…) some project, something to be included, some sort of project for boys or, or something…* (I 2)
Resources promoting mental health	Enough time	*(…) I would like to see fewer adolescents (in school health care). Then, when we are familiar with them, we are able to deal with them…* (I 2)
	Increased cooperation with others	*(…) interprofessional co-operation is extremely important, of course, that a curator and a psychologist are available…* (I 1)
